# Book Reviews

**Published:** 1917-07

**Authors:** 


### BOOK REVIEWS

Books mentioned may be inspected at and ordered through this office. So far as possible, books received in any month will be reviewed in the issue of the second month following. Pamphlets, quarterly and similar periodicals, reports, transactions, etc., will, as a rule, merely be mentioned.

### Annual Report, Dept. of Health, Buffalo, 1916.

This reflects great credit on the Commissioner, Dr. Francis Fronczak and his staff. The work of the Dept. is surprisingly comprehensive, even to physicians who should study the report carefully. To illustrate, we select various items at random. Dental services: 19,354, including 8,159 fillings and 2,561 extractions; visits to midwives' confinements 4,934; health center services, 69,136, including 37,785 at the centers, the remainder at hospitals and miscellaneous; expense of dept.: $222,864, including $181,700 salaries. 2,371 packages of free antityphoid vaccine were distributed to physicians, of which reports have been received from only 115. Of 210 bacteriologic examinations of sore eyes in babies, 87 were negative and only 10 revealed gonococci—an interesting result in view of the claims made of the frequency of this infection in the production of blindness. Total deaths 7,499 (14.77:1000) of which 757 were due to tuberculosis (almost exactly the classic 10% of all deaths), 99 being non-pulmonary. 466 deaths were due to cancer, 51 to typhoid. 492 deaths were due to accident and violence, including 40 suicides and 28 homicides. 148 deaths were due to methods of transit, including 65 from railroads, 57 from automobiles, 8 from wagons, 15 from trolleys, 2 from motorcycles and 1 from aeroplane. The decedents included 3,261 born in Buffalo, 1,491 born in other parts of the U. S., 1,026 born in Germany, the remainder being distributed among other foreign countries and 67 unknown. 2,753 deaths occurred in institutions. The tubercular death rate fluctuates considerably but averaged somewhat over 2% of the population up to 1891 and has ranged from 1.14-1.40 since 1898, the last named percentage being that for 1916. The tap water has varied in bacterial count per c.c. from 3 to 6700, colon bacilli being found in 1 c.c. on 9 days in Jan. 1916.

**The Practical Medicine Series,** Chas. L. Mix, A. M., M. D., Editor. Vol. 1, Series of 1917, General Medicine, Frank Billings, M. S., M. D. and Burrell O. Raulston, A. B., M. D., Editors, 383 pages, illustrated, $1.50. Price for Series of 10 volumes, $10. The Year Book Publishers, Chicago.

This year's series is bound in green to distinguish from previous years. The high standard of this review of current literature is maintained. The influence of the war is shown even in regard to distinctly medical conditions.

---

**University of Buffalo Bulletin.** Dept. of Arts and Sciences, April, 1917, Food Preparedness by A. P. Sy, Ph. D.

This is intended for popular education and contains a summary of dietetic chemistry, a number of recipes, and various practical hints as to economy.

---

**The Medical Clinics of Chicago,** May, 1917, No. 6, Vol. 2. Published bi-monthly by the W. B. Saunders Co., Philadelphia. $8 per year.

This number completes the volume and the next issue, July, 1917, will begin under a broader title: The Medical Clinics of N. America, bi-monthly as before, each number to consist of about 300 pages and to be limited to one city. The July issue will be devoted to the work of the Johns Hopkins Hospital and the Sept. issue to Philadelphia. N. Y., Boston and Chicago numbers will follow.

---

**Impotency, Sterility and Artificial Impregnation.** Frank P. Davis, Ph. B., M. D., published by C. V. Mosby Co., St. Louis, 138 pages, $1.25.

The author enters to a considerable degree into sexual psychology and literature and customs which stimulate the sexual desire. The last part of the work is especially valuable and includes aphroidisiac drugs and methods tending to increase the probability of conception, short of the direct introduction of semen into the uterus.

---

**Accuracy of Certified Causes of Death.** In our review in the May issue, we incorrectly stated that the committee was under the auspices of the Statistic Bureau of the Metropolitan Life Insurance Co., inferring this from the identity of address for mail.

